# RNA-Seq Reveals the Expression Profiles of Long Non-Coding RNAs in Lactating Mammary Gland from Two Sheep Breeds with Divergent Milk Phenotype

**DOI:** 10.3390/ani10091565

**Published:** 2020-09-03

**Authors:** Zhiyun Hao, Yuzhu Luo, Jiqing Wang, Jiang Hu, Xiu Liu, Shaobin Li, Xiayang Jin, Na Ke, Mengli Zhao, Liyan Hu, Yujie Lu, Xinmiao Wu, Lirong Qiao

**Affiliations:** Gansu Key Laboratory of Herbivorous Animal Biotechnology, Faculty of Animal Science and Technology, Gansu Agricultural University, Lanzhou 730070, China; haozy2018@163.com (Z.H.); luoyz@gsau.edu.cn (Y.L.); huj@gsau.edu.cn (J.H.); liux@gsau.edu.cn (X.L.); lisb@gsau.edu.cn (S.L.); jinxy@st.gsau.edu.cn (X.J.); ken@st.gsau.edu.cn (N.K.); 18394187234@163.com (M.Z.); huliyan2020@163.com (L.H.); luyujie1113@163.com (Y.L.); wuxinmiao2020@163.com (X.W.); qiaolr735757@163.com (L.Q.)

**Keywords:** long non-coding RNA, mammary gland, RNA-Seq, sheep

## Abstract

**Simple Summary:**

Long non-coding RNAs (lncRNAs) play a key role in regulating the expression level of mRNAs. The expression profiles of ovine mammary gland were investigated in two sheep breeds with divergent milk phenotype using RNA-Seq. A total of 1894 lncRNAs were found to be expressed and 68 of these were differentially expressed between the two breeds. Some important Gene Ontogeny (GO) terms and Kyoto Encyclopedia of Genes and Genomes (KEGG) pathways that were related to lactation and mammary gland morphogenesis were found for the target genes of differentially expressed lncRNAs. This study can improve our understanding of the functions of lncRNAs in the regulation of lactation, milk yield, and milk components in sheep.

**Abstract:**

Long non-coding RNAs (lncRNAs) are a kind of non-coding RNA with >200 nucleotides in length. Some lncRNAs have been proven to have clear regulatory functions in many biological processes of mammals. However, there have been no reports on the roles of lncRNAs in ovine mammary gland tissues. In the study, the expression profiles of lncRNAs were studied using RNA-Seq in mammary gland tissues from lactating Small-Tailed Han (STH) ewes and Gansu Alpine Merino (GAM) ewes with different milk yield and ingredients. A total of 1894 lncRNAs were found to be expressed. Compared with the GAM ewes, the expression levels of 31 lncRNAs were significantly up-regulated in the mammary gland tissues of STH ewes, while 37 lncRNAs were remarkably down-regulated. Gene Ontogeny (GO) enrichment and Kyoto Encyclopedia of Genes and Genomes (KEGG) analysis found that the target genes of differentially expressed lncRNAs were enriched in the development and proliferation of mammary epithelial cells, morphogenesis of mammary gland, ErbB signaling pathway, and Wnt signaling pathway. Some miRNA sponges of differentially expressed lncRNAs, reported to be associated with lactation and mammary gland morphogenesis, were found in a lncRNA-miRNA network. This study reveals comprehensive lncRNAs expression profiles in ovine mammary gland tissues, thereby providing a further understanding of the functions of lncRNAs in the lactation and mammary gland development of sheep.

## 1. Introduction

Long non-coding RNAs (lncRNAs) are a type of non-coding RNA that have more than 200 nucleotides [1]. At first, lncRNAs were regarded as ‘transcription noise’, hence they were ignored for a long time [2]. It is now more widely accepted that lncRNA plays a key role in many life activities, including dose compensation effect and the regulation of epigenetic change and cell cycle [1,3].

The majority of lncRNAs were found to regulate the mRNAs expression, and thus lead to the change of phenotype in different ways [4]. For example, some lncRNAs can act as ‘sponges’ for microRNAs (miRNAs) to inhibit or relieve the repression of target mRNAs by miRNAs, with an accompanying increase in the expression level of mRNAs in mammals [5,6]. Alternatively, some lncRNAs are transcribed from enhancer regions of mRNAs and can enhance the expression levels of nearby genes in cis [7], while the others can bind and sequester transcription factors, thereby resulting in a reduction in their transcriptional activity [8,9]. Finally, lncRNAs can also be involved in chromatin modification by forming lncRNA-ribonucleoprotein complexes with ribonucleic acid proteins, and thus regulate the expression of the target genes [10,11].

It has been confirmed that lncRNAs play key roles in the development of mammary gland and lactation in mice [12], dairy cows [13,14,15,16,17], dairy goats [6,18], and sows [19]. For example, lncRNA Neat1 is a basal component of paraspeckle nuclear bodies; its genetic ablation led to abnormal morphogenesis of mammary gland and defects in lactation owing to a decreased ability of sustaining high proliferation rates of duct during lobular-alveolar development in mice [12]. The overexpression of lncRNA SRA enhanced proliferation of mammary gland, augmented the ductal side branching, and induced precocious alveolar differentiation in the mammary gland of transgenic mice [20]. LncRNAs mPINC and Zfas1 were found to have growth-suppressive roles in mammary epithelial cells [21,22], in that knockout of either mPINC or Zfas1 in HC11 cells treated with lactogenic hormones enhanced lactogenic differentiation, and knockout of Zfas1 increased the proliferation rate of cells and induced the expression of *CSN2* in mice [21,22].

RNA sequencing (RNA-Seq) found some differentially expressed lncRNAs in mammary gland tissues between different periods. The functions of these differentially expressed lncRNAs can be achieved by targeting mRNAs. For example, 800 differentially expressed lncRNAs were found in the mammary gland of dairy goats between different lactation stages, and the target genes of these differentially expressed lncRNAs were enriched in multi-organism processes, reproductive processes and growth, and responses to stimuli and development that are closely correlated to the lactation and development of the mammary gland [18]. Yang et al. [16] found that target genes of differentially expressed lncRNAs in mammary gland tissues between the lactating and non-lactating Holstein cows, were significantly enriched in cell cycle, Janus kinase/signal transducer and activator of transcription (JAK-STAT), cell adhesion, and phosphatidylinositide 3-kinases protein kinase B (PI3K-Akt), and these signaling pathways were related to lactation.

Small-Tailed Han (STH) sheep and Gansu Alpine Merino (GAM) sheep are famous local breeds and are widely distributed in China. The two sheep breeds are of economic importance in the places in which they are raised. However, the two sheep breeds have different milk production performances. Overall, STH have higher milk yield. For example, the average milk yield of STH at 30 days postpartum is 1357 g/d. On the contrast, the milk yield of GAM is 853 g/d in the same period [23,24].

While the importance of lncRNAs in mammagenesis and lactation was well-studied in other domestic animals, to date, no studies have been reported about the expression profiles of lncRNAs in mammary gland tissues of any sheep breed. In this study, the expression profiles of lncRNAs were explored in the mammary gland tissues of lactating GAM ewes and STH ewes using RNA-Seq. Differentially expressed lncRNAs between the two breeds were obtained and their target genes were analyzed using Gene Ontogeny (GO) enrichment and Kyoto Encyclopedia of Genes and Genomes (KEGG). The results will contribute to better understanding the functions of lncRNAs in ovine mammagenesis and lactation.

## 2. Materials and Methods

### 2.1. Samples Collection

All sheep experiments were approved by Faculty of Animal Science and Technology, Gansu Agricultural University (Lanzhou, China) and were performed in agreement with the care and use guidelines of experimental animal published by the Ministry of Science and Technology of the People′s Republic of China (Approval number 2006-398).

Healthy, fourth-parity, three-year-old STH ewes (*n* = 9) and GAM ewes (*n* = 9) were selected for the investigation. These ewes were raised under the same environmental conditions that had natural light and free access to water and food in Songshan, Tian Zhu County (Gansu, China). The STH ewes have higher milk yield and content of protein and fat in milk than GAM ewes, and detailed data are shown in Supplementary File 1.

The parenchyma from the mammary gland tissue was collected by slaughtering all these ewes at lactation (21 days after lambing). The samples were dissected into 0.5 m^3^ cubes and immediately frozen in liquid nitrogen.

### 2.2. RNA Extraction

Total RNA was isolated and purified from mammary gland samples using Trizol Reagent (Life Technologies, Carlsbad, CA, USA). The concentration and quality of the RNA was detected using the NanoDrop 2000 (Thermo Scientific, Woltham, MA, USA). The RNA integrity number (RIN) was also assessed using the Agilent 2100 Bioanalyzer (Agilent Technologies, Santa Clara, CA, USA). Only the samples with RIN ≥7 were used for RNA-Seq.

### 2.3. Library Construction and RNA Sequencing

In order to reduce the effect of variation within individual ewes, the nine RNA samples isolated from each breed were randomly pooled to produce three samples in equal volume and concentration, as suggested by Hao et al. [23,24] and Paten et al. [25,26]. Briefly, the RNA isolated from three ewes in each breed was randomly pooled into a single sample. This brought about three separate sets of RNA from STH ewes and three sets of RNA from GAM ewes (i.e., six groups in total). The synthesis of cDNA and library construction was performed according to our previous study [24]. These six cDNA libraries (three from STH samples and three from GAM samples) were used for cluster generation with Illumina′s Cluster Station and then subjected to paired-end sequenced by Personal Corporation (Shanghai, China) [23,24].

### 2.4. Sequence Analysis

The quality of raw reads from the RNA-Seq was assessed using FastQC v0.10.1. Low-quality sequences and adaptor sequences and were removed to obtain clean reads from raw reads by Cutadapt v1.2.1. The clean reads for each library were mapped to the sheep reference genome v3.1 (Ensemble 9.2) using TopHat2 v2.0.9 [27].

### 2.5. Identification of lncRNA and Screening of Differentially Expressed lncRNAs

The alignment results from Tophat2 v2.0.9 were assembled to transcripts using Stringtie v1.2.4, and then the assembled transcripts were screened to obtain candidate lncRNAs based on the information of splicing results and the characteristics of lncRNAs [27]. Briefly, transcripts with <200 nucleotides and with a single exon were abandoned. The remaining transcripts were used to predict the coding potential using the Coding Potential Calculator [28], the Coding-Non-Coding Index [29], and the Protein Families Database [30] software, and the predicted results were intersected. These transcripts that would not encode protein from the above analysis were considered to be lncRNAs.

The expression level of lncRNA was normalized by calculating fragments per kilobase of transcript per million reads mapped (FPKM) using Stringtie v1.2.4. The lncRNAs with FPKM > 0.1 was considered to be meaningfully expressed. The DESeq v2.0 was used to analyze the differential expression of lncRNAs between the two breeds. These lncRNAs with |log2FoldChange| > 1 and *p*-value < 0.05 were considered as significant differentially expressed lncRNAs.

### 2.6. The Prediction of the Target Gene of lncRNAs and GO and KEGG Enrichment Analysis of the Target Genes

On the basis of the guidelines of the prediction of the target genes of lncRNAs, a gene closest to lncRNA within 100 kb was regarded as a target gene of the lncRNA [14]. The target genes of differentially expressed lncRNAs identified in the study were mapped to each term in the GO database, the number of target genes for each term was counted, and the hypergeometric distribution was performed to select significantly enriched GO terms. KOBAS v3.0 was used to perform KEGG pathway annotation, and the hypergeometric distribution was used to select significantly enriched pathways. The pathways with *p*-value < 0.05 were considered to be significantly enriched.

### 2.7. Validation of Differentially Expressed lncRNAs

To validate the repeatability and reliability of the lncRNA expression data obtained from RNA-Seq, nine differentially expressed lncRNAs were randomly selected to perform reverse transcription-quantitative PCR (RT-qPCR) using the individual RNA samples extracted originally for RNA-Seq. *β-actin* was chosen as a house-keeping reference gene [15]. The primer information of these differentially expressed lncRNAs and *β-actin* is listed in Supplementary File 2.

SuperScriptTM II reverse transcriptase (Invitrogen, Carlsbad, CA, USA) was used to synthesize the cDNA and the 2×ChamQ SYBR qPCR Master Mix (Vazyme, Nanjing, China) was used to perform RT-qPCR in triplicate. The relative expression levels of the lncRNAs were calculated using the 2^−ΔΔCt^ method. The RT-qPCR amplicons were detected by electrophoresis in 1.5% agarose gels and then confirmed by DNA sequencing.

### 2.8. Construction of the lncRNA-miRNA Network

Given that lncRNAs can play roles in the regulation of biological processes by functioning as miRNA sponges, the target miRNA sponges of differentially expressed lncRNAs were analyzed based on ovine miRbase v21.0 using miRanda v3.3a. A lncRNA-miRNA interaction network was analyzed using starbase v2.0 and further visualized using Cytoscape v3.3.0.

## 3. Results

### 3.1. Identification of lncRNAs in the Ovine Mammary Gland

**Figure 1:**
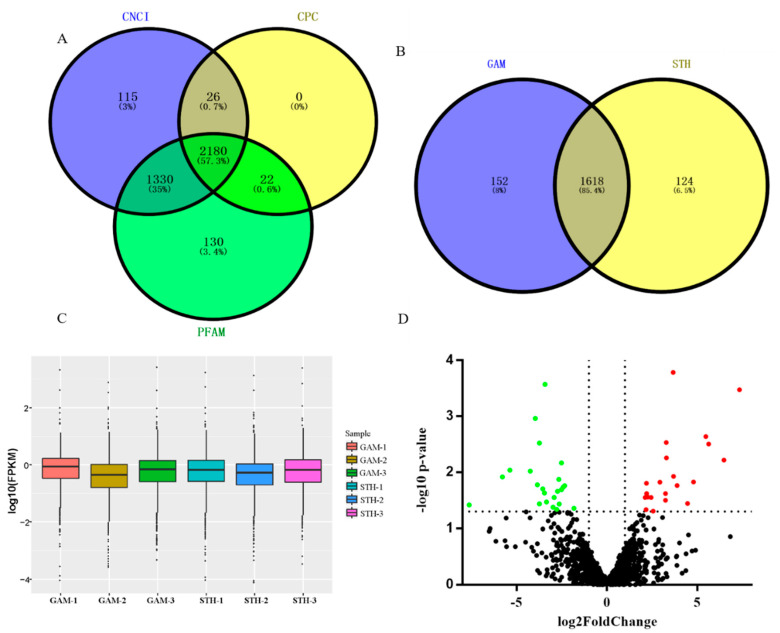
The characteristics analysis of lncRNAs obtained from RNA-Seq. (**A**) Venn diagram analyzing the coding potential analysis using Coding Potential Calculator (CPC), Coding-Non-Coding Index (CNCI), and Protein Families Database (PFAM). (**B**) Venn diagram summarizing the number of lncRNAs expressed only in the Small-Tailed Han (STH) ewes, expressed only in the Gansu Alpine Merino (GAM) ewes, and common to both groups. (**C**) The FPKM density distribution of lncRNA expression in sheep mammary gland between GAM and STH ewes. (**D**) Volcano plot comparing the change in lncRNAs’ expression of the STH and GAM ewes. The red and green dots indicate up-regulated and down-regulated lncRNAs in mammary gland of STH compared with the mammary gland of GAM, respectively (*p* < 0.05). The black dots represent lncRNAs that are not significantly different in the two breeds of sheep (*p* > 0.05).

For these pooled samples from mammary gland tissues of lactating STH and GAM ewes, their clean reads and mapped results to the ovine reference genome have been described in our previous study [23]. A total of 7201 transcripts were obtained in the study. After removing transcripts with less than 200 bp and transcripts with a single exon, an average of 3928 transcripts were found. The coding potential of these transcripts was analyzed and 2180 lncRNAs were then screened (Figure 1A).

Using a cut-off of FPKM > 0.1 to define expressed lncRNAs, a total of 1770 and 1742 lncRNA were detected in ovine mammary gland tissues of GAM and STH ewes, respectively (Figure 1B). Of all the lncRNAs detected, 1618 lncRNAs (85.43%) were expressed in both breeds (Figure 1B). Compared with the expression level of all lncRNAs identified in the study, it was found that the expression of MSTRG.75623.1 was the most abundant in both GAM and STH ewes.

The FPKM density distribution of lncRNA expression in ovine mammary gland indicated that most lncRNAs were expressed at low levels (Figure 1C).

### 3.2. Analysis and Validation of Differentially Expressed lncRNAs

According to the |log2foldchange| > 1 and the *p*-value < 0.05, 68 differentially expressed lncRNAs were identified in mammary gland tissues between the GAM and STH ewes (Supplementary File 3). Compared with the mammary gland tissues of GAM ewes, the expression levels of 31 lncRNAs were up-regulated in the mammary gland tissues of STH ewes, while 37 lncRNAs were down-regulated (Figure 1D).

In the mammary gland of STH ewes, the most prominent up-regulated lncRNAs were MSTRG.95989.3, MSTRG.67542.1, and MSTRG.61352.2, while the most prominent down-regulated lncRNAs were MSTRG.32232.1, MSTRG.91173.1, and MSTRG.42517.3.

### 3.3. Validation of Differentially Expressed lncRNAs Using RT-qPCR

**Figure 2:**
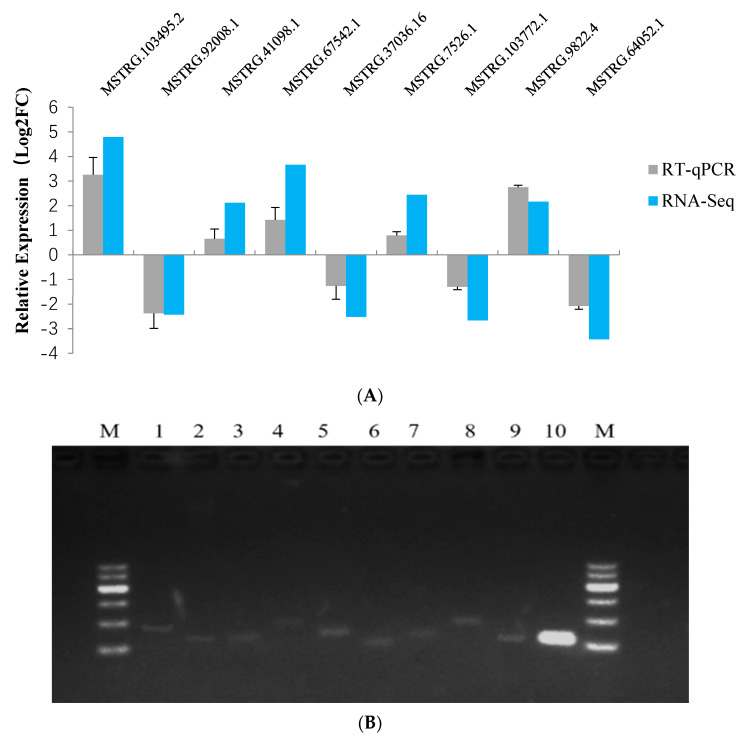
(**A**) RT-qPCR validation of nine differentially expressed lncRNAs identified using RNA-Seq. These result data showed the mean ±SD for three independent biological replicates and error bars indicated standard deviation. (**B**) RT-PCR amplification of lncRNAs using specific primers. 1: MSTRG.103495.2; 2: MSTRG.92008.1; 3: MSTRG.41098.1; 4: MSTRG.67542.1; 5: MSTRG.37036.16; 6: MSTRG.7526.1; 7: MSTRG.103772.1; 8: MSTRG.9822.4; 9: MSTRG.64052.1; 10: *β-actin*; M: marker (M1100, Solarbio, China), lanes represents 100–600 bp.

The RT-qPCR results showed that the expression levels of MSTRG.103495.2, MSTRG.41098.1, MSTRG.67542.1, MSTRG.7526.1, and MSTRG.9822.4 were up-regulated in the mammary gland tissues of STH ewes, while the expression levels of MSTRG.92008.1, MSTRG.37036.16, MSTRG.103772.1, and MSTRG.64052.1 were down-regulated (Figure 2A). These results suggest that the expression levels of the lncRNAs investigated by RT-qPCR were identical to those from RNA-Seq.

RT-qPCR amplicons of the nine differentially expressed lncRNAs were visualized by electrophoresis in 1.5% agarose gel (Figure 2B) and then confirmed by DNA sequencing. The results showed that PCR amplification using specific primer (Supplementary File 2) produced lncRNAs amplicons of the expected size. Taken together, these results confirmed the authenticity of the lncRNAs identified in the study.

### 3.4. GO Enrichment and KEGG Pathway Analysis of Differentially Expressed lncRNAs

For the 68 differentially expressed lncRNAs, the target genes were mainly major in 424 biological process (GO-BP) terms, 58 cellular components (GO-CC) terms, and 85 molecular function (GO-MF) terms (Supplementary File 4). Of all the GO-biological process (BP) terms enriched for the target genes of 68 differentially expressed lncRNAs found in the study, some important terms were found, including the development and proliferation of mammary epithelial cells and the morphogenesis of mammary gland (Table 1).

For the 68 differentially expressed lncRNAs, the target genes were majorly enriched in 39 signaling pathway (Supplementary File 4). Of these, the ErbB signaling pathway and wingless-type MMTV integration site family member (Wnt) signaling pathway were significantly enriched (Table 1).

### 3.5. Bioinformatic Analysis of lncRNA-miRNA Network

**Figure 3:**
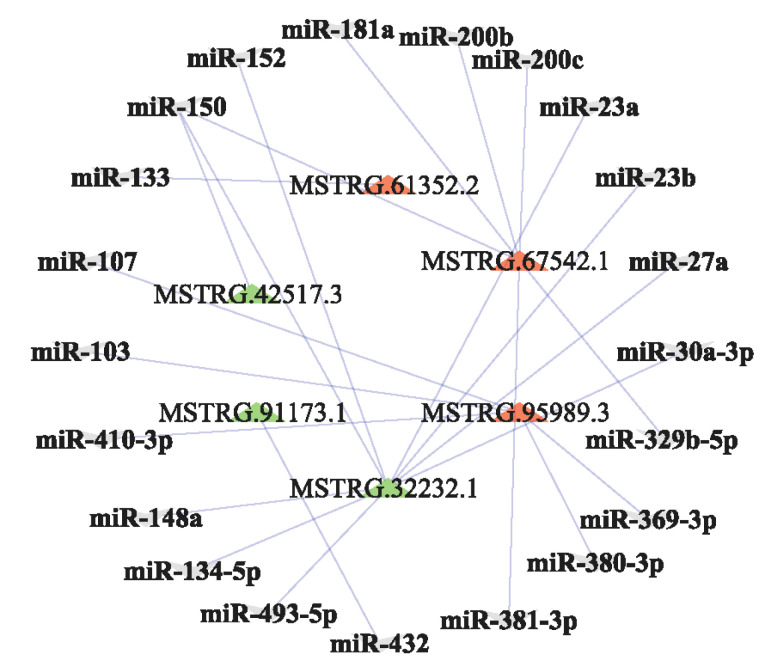
The lncRNA-miRNA interaction network. The red and green triangles represent up-regulated and down-regulated lncRNAs in the mammary gland of STH ewes compared with the mammary gland of GAM ewes, respectively. The gray inverted triangles represent the predicted target miRNA sponges of lncRNAs.

The function of lncRNAs could be achieved by targeting miRNAs [4,5,6]. The top 10 up-regulated and down-regulated lncRNAs and their target miRNA sponges are listed in Supplementary File 5. In order to improve the visibility of the results, the three most prominent up-regulated lncRNAs (MSTRG.95989.3, MSTRG.67542.1, and MSTRG.61352.2) and the three most prominent down-regulated lncRNAs (MSTRG.32232.1, MSTRG.91173.1, and MSTRG.42517.3) were selected to predict target miRNA sponges using miRanda v3.3a. For the six lncRNAs, a total of 217 pairs of the lncRNA–miRNA interaction were obtained, of which 21 target miRNAs with known function in previous literatures were screened to construct a lncRNA-miRNA network (Figure 3) [31,32,33,34,35]. Some target miRNA sponges of the six lncRNAs found in the study have been previously related with lactation and the development of mammary gland in dairy goats and cows [31,32,33,34]. For example, MSTRG. 32232.1 would target 16 miRNA sponges, among which miR-148a and miR-152 were found to be associated to lactation and the development of mammary gland development in dairy goats [31] and dairy cows [35].

## 4. Discussion

Although 98% of transcripts in mammalian genome are non-coding RNAs, it has now been proven that non-coding RNAs have important roles in the regulation of genes expression [36,37]. Given that lncRNAs are a widespread occurrence in eukaryote tissues, which account for 80% of the total number of non-coding RNAs [37], the roles of lncRNAs in various biological processes and cellular activities are worthy of further investigation and their study may contribute to our better understanding of molecular mechanisms underlying quantitative traits, including lactation and the development of the mammary gland in mammalian.

In the study, a total of 1770 and 1742 lncRNAs were found in mammary gland of lactating GAM and STH ewes, respectively. These were less than those reported in mammary gland tissue of dairy goats, with an average of 2381 predicted lncRNAs [18]. This likely reflects species-specific expression patterns, and the species-specific expression has been observed in the mammary gland of dairy cows and dairy goats [16,18]. Most lncRNAs identified in the study were expressed at low levels in the ovine mammary gland. This was consistent with previous studies reported from dairy cows by Zheng et al. [17] and dairy goats by Ji et al. [18], in which the expression of the most lncRNAs tends to be lower in mammary gland tissues.

Of all the lncRNAs identified in the study, the expression of MSTRG.75623.1 was the most abundant in both breeds. MSTRG.75623.1 is a 305 bp lncRNA located on chromosome 3 and would regulate *ASCL4*. *ASCL4* is a member of the bHLH (basic helix-loop-helix) transcription factor family, which is the family is related to the regulation of cell differentiation and cell proliferation in most mammalian tissues [38,39]. Although the role of *ASCL4* in the mammary gland has not yet been studied, another bHLH member of *Mist1* is exclusively expressed in lactating mammary epithelial cells of mice [40]. The loss of *Mist1* was found to impair the maintenance of the fully differentiated alveolar state in mouse mammary gland [40], indicating that *Mist1* plays a core role in regulating mammary gland cycle processes in the bHLH family. It was thus inferred that the high expression of MSTRG.75623.1 may be related to mammary gland development by regulating the expression of *ASCL4*. In order to show our results more clearly, the ten most expressed 10 lncRNAs, their target genes, and location identified in both STH and GAM ewes are shown in Supplementary File 6. However, the function of most lncRNAs and their target genes in mammary gland development and lactation needs further study.

MSTRG.67542.1 was one of the most prominent up-regulated lncRNAs in STH ewes in the study. Although the function of the lncRNA in mammary gland and lactation has not directly been studied, its role can be reflected by acting as miRNA sponges in the lncRNA-miRNA network (Figure 3). MSTRG.67542.1 would sponge miR-181a. The overexpression of miR-181a has been shown to decrease cellular lipid droplet synthesis in bovine mammary epithelial cells. On the contrary, the inhibition of miR-181a increased the concentration of lipid droplets by targeting *ACSL1* [41]. This suggests that MSTRG.67542.1 may promote the synthesis of lipid in milk by inhibiting miR-181a. This was identical to our observation that, compared with GAM ewes, MSTRG.67542.1 had a higher expression in STH ewes with a higher fat content in milk.

Of the 37 down-regulated lncRNAs in STH ewes found in the study, MSTRG.32232.1 was the most prominent down-regulated lncRNA. The same as MSTRG.67542.1, the role of MSTRG.32232.1 can also be reflected by its target miRNA sponges. MSTRG.32232.1 was found to potentially sponge miR-148a and miR-152 in the study (Figure 3). The overexpression miR-148a significantly accelerated the synthesis of triacylglycerol, while its knockdown prevented triacylglycerol synthesis by targeting *PPARGC1A* in goat mammary epithelial cells [31]. Meanwhile, the inhibition of miR-152 decreased milk fat synthesis by targeting *UCP3* [35]. Taken together, these results suggest that MSTRG.32232.1 may inhibit the synthesis of milk fat by targeting miR-148a and miR-152. This was in accordance with our observation that, compared with STH ewes, MSTRG.32232.1 had a higher expression in GAM ewes with a lower fat content in milk.

In order to further comprehend the function of differentially expressed lncRNAs between the STH and GAM ewes identified in the study, GO and KEGG analysis were performed for the target genes regulated by these differentially expressed lncRNAs (Table 1 and Supplementary File 4). Because the number of differentially expressed lncRNAs was low, the number of significantly enriched GO and KEGG terms was less too. For example, the target genes of MSTRG.23883.3 MSTRG.99147.2 and MSTRG.76074.1 were *CEBPB*, *ESR1*, and *PTHLH*, respectively (Table 1). *CEBPB* was involved in epithelial-mesenchymal transition, proliferation, and development of mammary epithelial cells in mouse [42]. The inhibition of *CEBPB* inhibited the levels of triacylglycerol and the formation of lipid droplet in bovine mammary epithelial cells [34]. The ESR1 protein is an essential transcription factor regulating ductal morphogenesis and the proliferation of mammary epithelial cells during postnatal periods in mice [43]. ESR1 has been shown to be involved in the development and proliferation of mammary gland epithelium cells, as well as gland morphogenesis in mice. The expression of *PTHLH* has been shown to have a critical role for the morphogenesis and angiogenesis of the mammary gland, and its expression contributed to mammary epithelial cells of mammals [44]. The three target genes were enriched in biological processes related to the development and proliferation of mammary gland epithelium cells, as well as mammary gland morphogenesis (Table 1). These suggest that the target genes of these lncRNAs play key roles in lactation and mammary gland morphogenesis.

The KEGG results showed that the target genes of MSTRG.100632.1, MSTRG.87828.1, and MSTRG.19103.1 were remarkably enriched in the ErbB signaling pathway and Wnt signaling pathway (Table 1). The ErbB signaling pathway plays important roles in postnatal mammary gland morphogenesis [45], in that it involved in mammary ductal outgrowth and morphogenesis in mice [46]. Canonical Wnt signaling accelerated mammary placode development, and it is also core in initiating the morphogenesis of the mammary gland [47]. These results suggest that these differentially expressed lncRNAs identified in the study may be involved in lactation and morphogenesis by targeting mRNAs.

## 5. Conclusions

This study describes the lncRNAs expression profiles of mammary gland of GAM and STH ewes. A total of 1894 lncRNAs were found to be expressed and 68 of these were differentially expressed between the two breeds with different milk performance. GO and KEGG analysis revealed that the target genes of differentially expressed lncRNAs were enriched in the development and proliferation of mammary epithelial cells, morphogenesis of mammary gland, ErbB signaling pathway, and Wnt signaling pathway. This study provides an improved understanding of the roles of lncRNAs in the mammary gland of sheep.

## Figures and Tables

**Table 1:** Functional annotation of the target genes of differentially expressed lncRNAs between Gansu Alpine Merino (GAM) and Small-Tailed Han (STH) ewes. KEGG, Kyoto Encyclopedia of Genes and Genomes; GO, Gene Ontogeny.

KEGG Pathway/GO Term	LncRNA	Target Genes	*p*-Value
KEGG: ErbB signaling pathway	MSTRG.100632.1|MSTRG.87828.1	*PTK2*|*CAMK2D*	*p* < 0.05
KEGG: Wnt signaling pathway	MSTRG.19103.1 |MSTRG.87828.1	*CACYBP*|*CAMK2D*	*p* < 0.05
GO: 0061180 mammary gland epithelium development	MSTRG.23883.3|MSTRG.99147.2 |MSTRG.76074.1	*CEBPB*|*ESR1*|*PTHLH*	*p* < 0.05
GO: 0033598 mammary gland epithelial cell proliferation	MSTRG.23883.3|MSTRG.99147.2	*CEBPB|ESR1*	*p* < 0.05
GO: 0060443 mammary gland morphogenesis	MSTRG.99147.2 |MSTRG.76074.1	*ESR1|PTHLH*	*p* < 0.05

## Supplementary Materials

The following are available online at https://www.mdpi.com/2076-2615/10/9/1565/s1, Supplementary File 1. Comparison of body weight, milk yield, and milk composition of Gansu Alpine Merino ewes with those of Small-Tailed Han ewes; Supplementary File 2. Primers used for RT-qPCR analyses; Supplementary File 3. The differentially expressed lncRNAs in the mammary gland tissues between STH and GAM ewes; Supplementary File 4. The information of GO and KEGG statistical analysis for differentially expressed lncRNAs in the mammary gland tissues between STH and GAM ewes; Supplementary File 5. The target miRNA sponges and genes of the top 10 up/down-regulated lncRNAs in the STH ewes; Supplementary File 6. The 10 most expressed lncRNAs identified in the mammary gland tissues of both STH and GAM ewes.
