# Supplementary material for: RNA-Seq Reveals the Expression Profiles of Long Non-Coding RNAs in Lactating Mammary Gland from Two Sheep Breeds with Divergent Milk Phenotype
Source: Animals (Basel). 2020 Sep 3;10(9):1565. doi: 10.3390/ani10091565 (PMC7552154; doi:10.3390/ani10091565)
Supplement: Supplementary file 1 [file animals-10-01565-s001.zip › Supplementary File 1.docx]

**Supplementary File 1.** Comparison of body weight, milk yield and milk composition of Gansu Alpine Merino ewes with those of Small-Tailed Han ewes ^1^

| Trait | Small tail Han Sheep | Gansu Alpine Merino sheep |
| --- | --- | --- |
| Body weight (kg) | 64.4±8.43 ^A^ | 40.0±6.25 ^B^ |
| Milk yield (g/d) | 1357±65.42 ^A^ | 853±55.38 ^B^ |
| Lactoprotein (%) | 5.37±0.11 ^A^ | 4.67±0.11 ^B^ |
| Casein (%) | 4.53±0.13 ^A^ | 3.92±0.06 ^B^ |
| Milk fat (%) | 7.42±1.51 ^A^ | 5.66±0.35 ^B^ |
| Lactose (%) | 4.74±0.21 | 4.85±0.11 |
| Milk solids (%) | 18.76±2.42 | 16.15±0.34 |
| Non-fat milk solids (%) | 11.24±0.30 | 10.64±0.14 |

^1^ Means within rows that do not share a superscript uppercase (A or B) are different at *p* < 0.01.
