# Supplementary material for: RNA-Seq Reveals the Expression Profiles of Long Non-Coding RNAs in Lactating Mammary Gland from Two Sheep Breeds with Divergent Milk Phenotype
Source: Animals (Basel). 2020 Sep 3;10(9):1565. doi: 10.3390/ani10091565 (PMC7552154; doi:10.3390/ani10091565)
Supplement: Supplementary file 1 [file animals-10-01565-s001.zip › Supplementary File 2.docx]

**Supplementary File 2.** Primers used for RT-qPCR analyses

| **lncRNA** | **Forward (5'→3')** | **Reverse (5'→3')** | **Product size (bp)** |
| --- | --- | --- | --- |
| MSTRG.103495.2 | CGTCGATTCCTCCTCATC | CCAGGCTCCTCTGTTCTT | 164 |
| MSTRG.92008.1 | TGTCCACCACCAAGTCAA | AGAGGAATCAGCCAGCAC | 116 |
| MSTRG.41098.1 | CACCTGGCTATTCAAACG | AGTCCATAGGGTCACAAA | 127 |
| MSTRG.67542.1 | GCCCAATCTTTAGGTCTG | GGACACGACTTAGCGACT | 196 |
| MSTRG.37036.16 | TCGGTTTATCTATTCATCAG | ACCCAGCAATTCTACTCCTA | 149 |
| MSTRG.7526.1 | ATGACATCTGGCATTTAG | CAGTTATGAAGACCCTCC | 99 |
| MSTRG.103772.1 | TGTCCACCAGTTGAGATT | ACAAGCGATGTAGTCAGG | 134 |
| MSTRG.9822.4 | CAGGAAAGGGTGAGGAGT | CGGGAACGAGACAAACTCAT | 191 |
| MSTRG.64052.1 | CCGCTTTAGCTTGGGTAG | GGCCTCCGTTTACTGATAGTC | 119 |
| β-actin | AGCCTTCCTTCCTGGGCATGGA | GGACAGCACCGTGTTGGCGTA | 113 |
