# Supplementary material for: RNA-Seq Reveals the Expression Profiles of Long Non-Coding RNAs in Lactating Mammary Gland from Two Sheep Breeds with Divergent Milk Phenotype
Source: Animals (Basel). 2020 Sep 3;10(9):1565. doi: 10.3390/ani10091565 (PMC7552154; doi:10.3390/ani10091565)
Supplement: Supplementary file 1 [file animals-10-01565-s001.zip › Supplementary File 3.docx]

| **Supplementary File 3.** The differentially expressed lncRNAs in the mammary gland tissues between STH and GAM ewes   \| **LncRNA** \| **BaseMeanA^1^** \| **BaseMeanB^2^** \| **FoldChange** \| **Log_2_FoldChange** \| ***p*-value** \| **Change^3^** \| **Target gene** \| \| --- \| --- \| --- \| --- \| --- \| --- \| --- \| --- \| \| MSTRG.95989.3 \| 516.2192651 \| 0 \| Inf^4^ \| Inf \| 3.20717E-05 \| Up \| *FOXN3* \| \| MSTRG.67542.1 \| 839.5711864 \| 66.1577454 \| 12.69044435 \| 3.66567068 \| 0.000163544 \| Up \| *SH2D4A* \| \| MSTRG.61352.2 \| 176.932952 \| 0 \| Inf \| Inf \| 0.000303891 \| Up \| *TRYPTASE-1* \| \| MSTRG.12659.4 \| 398.491038 \| 2.462539701 \| 161.8211629 \| 7.338256484 \| 0.000335031 \| Up \| *HLF* \| \| MSTRG.67509.2 \| 50.0115235 \| 0 \| Inf \| Inf \| 0.000422971 \| Up \| *FNTA* \| \| MSTRG.22606.2 \| 47.15778133 \| 0 \| Inf \| Inf \| 0.001028274 \| Up \| *GNAS* \| \| MSTRG.344.2 \| 215.0555372 \| 0 \| Inf \| Inf \| 0.001657726 \| Up \| *ENSOARG00000019151* \| \| MSTRG.70334.1 \| 91.15273654 \| 2.053588494 \| 44.38705067 \| 5.472066946 \| 0.002300249 \| Up \| *SLC1A4* \| \| MSTRG.43574.1 \| 216.2159322 \| 0 \| Inf \| Inf \| 0.002871139 \| Up \| *ENSOARG00000007799* \| \| MSTRG.83799.2 \| 299.4578317 \| 30.89215201 \| 9.693653963 \| 3.277040583 \| 0.002944289 \| Up \| *ENSOARG00000007856* \| \| MSTRG.31339.1 \| 198.7106188 \| 4.021339721 \| 49.41403427 \| 5.62684894 \| 0.003130775 \| Up \| *LCP2* \| \| MSTRG.39144.3 \| 965.154443 \| 0 \| Inf \| Inf \| 0.004364443 \| Up \| *EXOC3L4* \| \| MSTRG.87828.1 \| 301.5606598 \| 30.8386541 \| 9.778658263 \| 3.289636525 \| 0.005534646 \| Up \| *CAMK2D* \| \| MSTRG.27098.4 \| 63.76223153 \| 0.717872727 \| 88.82108086 \| 6.472830222 \| 0.00605898 \| Up \| *BCAM* \| \| MSTRG.90061.1 \| 75.2917101 \| 5.835808984 \| 12.90167487 \| 3.68948646 \| 0.011813264 \| Up \| *ENSOARG00000025179* \| \| MSTRG.103495.2 \| 350.2467058 \| 12.67748741 \| 27.62745443 \|  \| 0.014879289 \| Up \| *HERC6* \| \| MSTRG.19103.1 \| 471.4266202 \| 61.85283557 \| 7.621746293 \| 2.930121586 \| 0.014964308 \| Up \| *CACYBP* \| \| MSTRG.30356.1 \| 665.9109682 \| 146.1176971 \| 4.557360138 \| 2.188198382 \| 0.015691982 \| Up \| *GDPD4* \| \| MSTRG.98304.2 \| 134.363586 \| 9.09858618 \| 14.76752359 \| 3.884356011 \| 0.01717502 \| Up \| *MAP7* \| \| MSTRG.41118.1 \| 878.5527215 \| 0 \| Inf \| Inf \| 0.021200055 \| Up \| *ENSOARG00000014113* \| \| MSTRG.61259.1 \| 94.71947144 \| 9.998595843 \| 9.473277341 \| 3.243863622 \| 0.023838728 \| Up \| *MPG* \| \| MSTRG.23714.1 \| 759.5604984 \| 165.5194697 \| 4.588949565 \| 2.198163951 \| 0.023906522 \| Up \| *SLC2A10* \| \| MSTRG.13234.6 \| 292.7316287 \| 61.16646097 \| 4.785819287 \| 2.258765922 \| 0.02771559 \| Up \| *OMG* \| \| MSTRG.7526.1 \| 3721.898477 \| 682.792763 \| 5.450992862 \| 2.446519031 \| 0.02800685 \| Up \| *ATP11B* \| \| MSTRG.41098.1 \| 542.8258764 \| 124.9937782 \| 4.342823173 \| 2.118633212 \| 0.028171408 \| Up \| *PTPRG* \| \| MSTRG.6087.1 \| 68.28880087 \| 7.242699465 \| 9.428639307 \| 3.237049584 \| 0.031537762 \| Up \| *NFKBIZ* \| \| MSTRG.50528.2 \| 216.4492297 \| 9.814828965 \| 22.05328595 \| 4.462921729 \| 0.035995553 \| Up \| *ENSOARG00000019770* \| \| MSTRG.9822.4 \| 554.8185553 \| 123.6756775 \| 4.48607654 \| 2.165454235 \| 0.046224908 \| Up \| *KCNJ15* \| \| MSTRG.64038.5 \| 207.7536574 \| 0 \| Inf \| Inf \| 0.046432658 \| Up \| *PDGFA* \| \| MSTRG.83385.1 \| 200.0809371 \| 0 \| Inf \| Inf \| 0.047273678 \| Up \| *ENSOARG00000012799* \| \| MSTRG.99147.2 \| 191.2640273 \| 32.41313486 \| 5.900818545 \| 2.560915095 \| 0.049258736 \| Up \| *ESR1* \| \| MSTRG.32232.1 \| 0 \| 995.7194547 \| 0 \| -Inf \| 1.40204E-06 \| Down \| *HSPB3* \| \| MSTRG.91173.1 \| 0 \| 61.1123632 \| 0 \| -Inf \| 9.4694E-05 \| Down \| *CCDC96* \| \| MSTRG.42517.3 \| 0 \| 534.6935133 \| 0 \| -Inf \| 0.000206474 \| Down \| *ENSOARG00000004795* \| \| MSTRG.95989.2 \| 0.370835358 \| 406.7727298 \| 0.000911652 \| -10.09922844 \| 0.000250841 \| Down \| *FOXN3* \| \| MSTRG.64052.1 \| 127.8384967 \| 1377.292323 \| 0.09281871 \| -3.429440542 \| 0.000267853 \| Down \| *UNCX* \| \| MSTRG.88204.2 \| 10.0484197 \| 157.2902505 \| 0.063884568 \| -3.968388716 \| 0.001094048 \| Down \| *UBE2D3* \| \| MSTRG.15907.3 \| 0 \| 87.32420552 \| 0 \| -Inf \| 0.002653119 \| Down \| *MRPL38* \| \| MSTRG.75667.3 \| 550.6905066 \| 7351.182814 \| 0.074911823 \| -3.738662757 \| 0.003018024 \| Down \| *LARGE* \| \| MSTRG.53145.1 \| 0 \| 127.5991183 \| 0 \| -Inf \| 0.006221646 \| Down \| *ENSOARG00000009395* \| \| MSTRG.37036.16 \| 1638.806793 \| 9387.059614 \| 0.174581483 \| -2.518027544 \| 0.006809082 \| Down \| *MFGE8* \| \| MSTRG.19900.1 \| 0.994871793 \| 41.03258721 \| 0.024245895 \| -5.366115692 \| 0.009149341 \| Down \| *DDX59* \| \| MSTRG.66946.3 \| 58.6187233 \| 1112.983702 \| 0.052668088 \| -4.24692711 \| 0.009504136 \| Down \| *RBPMS* \| \| MSTRG.85066.1 \| 0.624036435 \| 34.46475798 \| 0.018106509 \| -5.78734781 \| 0.01207136 \| Down \| *ZFP62* \| \| MSTRG.103772.1 \| 85.33949226 \| 539.3722455 \| 0.158220029 \| -2.659995855 \| 0.013362456 \| Down \|  \| \| MSTRG.63876.1 \| 70.24041023 \| 432.5900529 \| 0.162371765 \| -2.622627316 \| 0.013648528 \| Down \| *AMZ1* \| \| MSTRG.77261.2 \| 0 \| 39.38495448 \| 0 \| -Inf \| 0.015655729 \| Down \| *TEAD4* \| \| MSTRG.79806.1 \| 55.25446834 \| 798.1335821 \| 0.0692296 \| -3.852467185 \| 0.016634487 \| Down \| *NAMPT* \| \| MSTRG.71740.1 \| 104.5662596 \| 528.3726314 \| 0.19790249 \| -2.337138328 \| 0.017288154 \| Down \|  \| \| MSTRG.92008.1 \| 45.24191368 \| 242.9671495 \| 0.186205887 \| -2.425029407 \| 0.018337836 \| Down \| *SERINC5* \| \| MSTRG.82051.1 \| 4.450024296 \| 52.11916667 \| 0.085381724 \| -3.549928899 \| 0.019751763 \| Down \| *KIAA1147* \| \| MSTRG.65717.10 \| 249.1826948 \| 1425.713489 \| 0.174777539 \| -2.516408303 \| 0.020364595 \| Down \| *ENSOARG00000007679* \| \| MSTRG.32020.2 \| 0 \| 1297.611012 \| 0 \| -Inf \| 0.020604769 \| Down \| *PDE4D* \| \| MSTRG.52863.1 \| 35.41499411 \| 235.7410898 \| 0.150228346 \| -2.734771036 \| 0.02171334 \| Down \| *TNFRSF21* \| \| MSTRG.41120.2 \| 0 \| 660.2364933 \| 0 \| -Inf \| 0.023118045 \| Down \| *ENSOARG00000014113* \| \| MSTRG.22606.1 \| 7.377615674 \| 81.21383655 \| 0.090841856 \| -3.460499002 \| 0.023319693 \| Down \| *ENSOARG00000004809* \| \| MSTRG.54158.3 \| 0 \| 21.89809809 \| 0 \| -Inf \| 0.025320483 \| Down \| *RREB1* \| \| MSTRG.100632.1 \| 21.67112039 \| 163.6526465 \| 0.132421448 \| -2.916791286 \| 0.028076809 \| Down \| *PTK2* \| \| MSTRG.42633.1 \| 0 \| 18.79126287 \| 0 \| -Inf \| 0.033605958 \| Down \| *TMEM18* \| \| MSTRG.56367.1 \| 7.022416911 \| 71.13175411 \| 0.098724079 \| -3.340454186 \| 0.033799576 \| Down \| *RNASEH2C* \| \| MSTRG.73341.1 \| 3.246150897 \| 43.22484001 \| 0.0750992 \| -3.735058654 \| 0.036542779 \| Down \| *TMCC3* \| \| MSTRG.91073.1 \| 14.82337513 \| 92.35438404 \| 0.160505376 \| -2.639306476 \| 0.036857374 \| Down \| *BST1* \| \| MSTRG.75667.2 \| 7.488437218 \| 1477.074324 \| 0.005069777 \| -7.623862037 \| 0.038208511 \| Down \| *LARGE* \| \| MSTRG.105947.3 \| 294.0797768 \| 2322.949595 \| 0.126597571 \| -2.981678368 \| 0.041881808 \| Down \| *CYTB* \| \| MSTRG.23883.3 \| 0 \| 23.28535828 \| 0 \| -Inf \| 0.043359187 \| Down \| *CEBPB* \| \| MSTRG.68472.1 \| 130.7846868 \| 462.3533334 \| 0.282867403 \| -1.82180216 \| 0.043748442 \| Down \| *PHYHD1* \| \| MSTRG.65192.1 \| 9.27088395 \| 63.26181921 \| 0.146547856 \| -2.770556237 \| 0.045982898 \| Down \| *SLC29A3* \| \| MSTRG.76074.1 \| 0 \| 129.3115898 \| 0 \| -Inf \| 0.046582879 \| Down \| *PTHLH* \| |
| --- | --- | --- | --- | --- | --- | --- | --- | --- | --- | --- | --- | --- | --- | --- | --- | --- | --- | --- | --- | --- | --- | --- | --- | --- | --- | --- | --- | --- | --- | --- | --- | --- | --- | --- | --- | --- | --- | --- | --- | --- | --- | --- | --- | --- | --- | --- | --- | --- | --- | --- | --- | --- | --- | --- | --- | --- | --- | --- | --- | --- | --- | --- | --- | --- | --- | --- | --- | --- | --- | --- | --- | --- | --- | --- | --- | --- | --- | --- | --- | --- | --- | --- | --- | --- | --- | --- | --- | --- | --- | --- | --- | --- | --- | --- | --- | --- | --- | --- | --- | --- | --- | --- | --- | --- | --- | --- | --- | --- | --- | --- | --- | --- | --- | --- | --- | --- | --- | --- | --- | --- | --- | --- | --- | --- | --- | --- | --- | --- | --- | --- | --- | --- | --- | --- | --- | --- | --- | --- | --- | --- | --- | --- | --- | --- | --- | --- | --- | --- | --- | --- | --- | --- | --- | --- | --- | --- | --- | --- | --- | --- | --- | --- | --- | --- | --- | --- | --- | --- | --- | --- | --- | --- | --- | --- | --- | --- | --- | --- | --- | --- | --- | --- | --- | --- | --- | --- | --- | --- | --- | --- | --- | --- | --- | --- | --- | --- | --- | --- | --- | --- | --- | --- | --- | --- | --- | --- | --- | --- | --- | --- | --- | --- | --- | --- | --- | --- | --- | --- | --- | --- | --- | --- | --- | --- | --- | --- | --- | --- | --- | --- | --- | --- | --- | --- | --- | --- | --- | --- | --- | --- | --- | --- | --- | --- | --- | --- | --- | --- | --- | --- | --- | --- | --- | --- | --- | --- | --- | --- | --- | --- | --- | --- | --- | --- | --- | --- | --- | --- | --- | --- | --- | --- | --- | --- | --- | --- | --- | --- | --- | --- | --- | --- | --- | --- | --- | --- | --- | --- | --- | --- | --- | --- | --- | --- | --- | --- | --- | --- | --- | --- | --- | --- | --- | --- | --- | --- | --- | --- | --- | --- | --- | --- | --- | --- | --- | --- | --- | --- | --- | --- | --- | --- | --- | --- | --- | --- | --- | --- | --- | --- | --- | --- | --- | --- | --- | --- | --- | --- | --- | --- | --- | --- | --- | --- | --- | --- | --- | --- | --- | --- | --- | --- | --- | --- | --- | --- | --- | --- | --- | --- | --- | --- | --- | --- | --- | --- | --- | --- | --- | --- | --- | --- | --- | --- | --- | --- | --- | --- | --- | --- | --- | --- | --- | --- | --- | --- | --- | --- | --- | --- | --- | --- | --- | --- | --- | --- | --- | --- | --- | --- | --- | --- | --- | --- | --- | --- | --- | --- | --- | --- | --- | --- | --- | --- | --- | --- | --- | --- | --- | --- | --- | --- | --- | --- | --- | --- | --- | --- | --- | --- | --- | --- | --- | --- | --- | --- | --- | --- | --- | --- | --- | --- | --- | --- | --- | --- | --- | --- | --- | --- | --- | --- | --- | --- | --- | --- | --- | --- | --- | --- | --- | --- | --- | --- | --- | --- | --- | --- | --- | --- | --- | --- | --- | --- | --- | --- | --- | --- | --- | --- | --- | --- | --- | --- | --- | --- | --- | --- | --- | --- | --- | --- | --- | --- | --- | --- | --- | --- | --- | --- | --- | --- | --- | --- | --- | --- | --- | --- | --- | --- | --- | --- | --- | --- | --- | --- | --- | --- | --- | --- | --- | --- | --- | --- | --- | --- | --- | --- | --- | --- | --- | --- | --- | --- | --- | --- | --- | --- | --- | --- | --- | --- | --- | --- | --- | --- | --- | --- | --- | --- | --- | --- |

^1^ BaseMean is used to homogenize the expression of the gene in both the total sample and the single sample in the DEGSeq R package (Wang et al., 2010). BaseMean A is the BaseMean value in mammary gland of STH ewes.

^2^ BaseMean B is the BaseMean value in mammary gland of GAM ewes.

^3^ The difference in the expression of a specific lncRNA in the mammary gland tissue of STH ewes, compared to the mammary gland of GAM ewes.

^4^ Represents infinity.
