# Supplementary material for: RNA-Seq Reveals the Expression Profiles of Long Non-Coding RNAs in Lactating Mammary Gland from Two Sheep Breeds with Divergent Milk Phenotype
Source: Animals (Basel). 2020 Sep 3;10(9):1565. doi: 10.3390/ani10091565 (PMC7552154; doi:10.3390/ani10091565)
Supplement: Supplementary file 1 [file animals-10-01565-s001.zip › Supplementary File 6.docx]

| **LncRNA** | **BaseMeanA** | **BaseMeanB** | **Location** | **Target Gene** |
| --- | --- | --- | --- | --- |
| MSTRG.75623.1 | 71343.08159 | 75390.70619 | Chr9:75283583-75283771 | *ASCL4* |
| MSTRG.96198.1 | 53762.81033 | 30628.58198 | Chr7:99922927-99926721 | *B2M* |
| MSTRG.104253.1 | 36473.23163 | 38670.05312 | JH921799.1:7541-9489 | *-* |
| MSTRG.75078.1 | 28787.46994 | 40571.1683 | Chr3:163139331-163139708 | *ZC3H10* |
| MSTRG.81909.1 | 18954.18053 | 17281.20092 | Chr4:101361654-101365408 | *CREB3L2* |
| MSTRG.67687.2 | 23572.78912 | 8722.546953 | Chr26:39965168-39972085 | *ERBA, BETA1* |
| MSTRG.88906.10 | 9656.664345 | 13930.68162 | Chr6:45025459-45028429 | *SLC34A2* |
| MSTRG.1827.7 | 11273.52071 | 10418.8607 | Chr1:40132606-40143555 | *JAK1* |
| MSTRG.10961.4 | 8766.923796 | 9756.085051 | Chr10:27798110-27812673 | *RFC3* |
| MSTRG.26719.1 | 9531.293032 | 5975.933709 | Chr14:47604325-47619640 | *EIF3K* |

**Supplementary File 6**. The 10 most expressed lncRNAs identified in the mammary gland tissues of both STH and GAM ewes
